# *ERCC2 *2251A>C genetic polymorphism was highly correlated with early relapse in high-risk stage II and stage III colorectal cancer patients: A preliminary study

**DOI:** 10.1186/1471-2407-8-50

**Published:** 2008-02-12

**Authors:** Ming-Yii Huang, Wei-Yu Fang, Su-Chen Lee, Tian-Lu Cheng, Jaw-Yuan Wang, Shiu-Ru Lin

**Affiliations:** 1 Graduate Institute of Medicine, Kaohsiung Medical University, Kaohsiung, Taiwan; 2 Department of Radiation Oncology, Kaohsiung Medical University Hospital, Kaohsiung, Taiwan; 3 Department of Radiation Oncology, Faculty of Medicine, College of Medicine, Kaohsiung Medical University, Kaohsiung, Taiwan; 4 Graduate Institute of Medical Genetics, Kaohsiung Medical University, Kaohsiung, Taiwan; 5 Laboratory Medicine, Kaohsiung Medical University Hospital, Kaohsiung, Taiwan; 6 Faculty of Biomedical Science and Environmental Biology, Kaohsiung Medical University, Kaohsiung, Taiwan; 7 Division of Gastrointestinal and General Surgery, Department of Surgery, Kaohsiung Medical University Hospital, Kaohsiung, Taiwan; 8 Department of Surgery, Faculty of Medicine, College of Medicine, Kaohsiung Medical University, Kaohsiung, Taiwan

## Abstract

**Background:**

Early relapse in colorectal cancer (CRC) patients is attributed mainly to the higher malignant entity (such as an unfavorable genotype, deeper tumor invasion, lymph node metastasis and advance cancer stage) and poor response to chemotherapy. Several investigations have demonstrated that genetic polymorphisms in drug-targeted genes, metabolizing enzymes, and DNA-repairing enzymes are all strongly correlated with inter-individual differences in the efficacy and toxicity of many treatment regimens. This preliminary study attempts to identify the correlation between genetic polymorphisms and clinicopathological features of CRC, and evaluates the relationship between genetic polymorphisms and chemotherapeutic susceptibility of Taiwanese CRC patients. To our knowledge, this study discusses, for the first time, early cancer relapse and its indication by multiple genes.

**Methods:**

Six gene polymorphisms functional in drug-metabolism – *GSTP1 *Ile105Val, *ABCB1 *Ile1145Ile, *MTHFR *Ala222Val, *TYMS *double (2R) or triple (3R) tandem repeat – and DNA-repair genes – *ERCC2 *Lys751Gln and *XRCC1 A*rg399Gln – were assessed in 201 CRC patients using a polymerase chain reaction-restriction fragment-length polymorphism (PCR-RFLP) technique and DNA sequencing. Patients were diagnosed as either high-risk stage II (T2 and 3 N0 M0) or III (any T N1 and 2 M0) and were administered adjuvant chemotherapy regimens that included 5-fluorouracil (5FU) and leucovorin (LV). The correlations between genetic polymorphisms and patient clinicopathological features and relapses were investigated.

**Results:**

In this study, the distributions of *GSTP1 *(*P *= 0.003), *ABCB1 *(*P *= 0.001), *TYMS *(*P *< 0.0001), *ERCC2 *(*P *< 0.0001) and *XRCC1 *(*P *= 0.006) genotypes in the Asian population, with the exception of *MTHFR *(*P *= 0.081), differed significantly from their distributions in a Caucasian population. However, the unfavorable genotype *ERCC2 *2251A>C (*P *= 0.006), tumor invasion depth (*P *= 0.025), lymph node metastasis (*P *= 0.011) and cancer stage (*P *= 0.008) were significantly correlated with early relapse. Patients carrying the *ERCC2 *2251AC or2251CC genotypes had a significantly increased risk of early relapse (OR = 3.294, 95% CI, 1.272–8.532).

**Conclusion:**

We suggest that *ERCC2 *2251A>C alleles may be genetic predictors of early CRC relapse.

## Background

The primary treatment for colorectal cancer (CRC) is resection of the primary tumor. After surgery, patients are frequently administered adjuvant chemotherapy to eliminate cancer cells that may have metastasized [1]. Despite chemotherapy, CRC remains the third major cause of cancer-related death in Taiwan, accounting for >3,000 deaths per year [2]. The overall five-year survival is 50–60% in European countries [3], a result similar to that in Taiwan [4]. The primary cause of death is distant and loco-regional relapses. Notably, CRC relapse is strongly correlated with chemotherapeutic drug response [1,5,6].

Genetic polymorphisms in drug-targeted genes [7,8], metabolizing enzymes [9], and DNA-repairing enzymes [10] have been linked to inter-individual differences in the efficacy and toxicity of numerous drugs. Several studies have investigated various gene expressions and chemotherapeutic drug responses of cancers. For instance, a polymorphic 28-bp tandem double repeat polymorphism in the regulatory region of the *TYMS *gene is correlated with a better response to 5-fluorouracil (5 FU) chemotherapy than the triple repeat in the polymorphism [11]. A common polymorphism in the *MTHFR *gene (677C>T; Ala222Val) increases the efficacy of fluoropyrimidine-based chemotherapy [12]. In Caucasian patients with advanced CRC who are treated with oxaliplatin, 5 FU and leucovorin (LV), *ERCC1 *118 T/T, *XRCC1 *(Arg – >Gln substitution in exon 10), *ERCC2 *751AC, and *ERCC2 *751CC genotypes are independently associated with poor progression-free survival and short-term survival [13,14]. Glutathione S-transferases (GSTs) participate in the detoxification of platinum compounds and are important mediators of intrinsic and acquired resistance to oxaliplatin [8].

Advanced CRC is one of the most chemotherapy-resistant human malignancies. The cytotoxic agent with the most consistent antitumor activity is 5 FU. However, significant variability in drug response can occur among cancer patients treated with the same medications [15]. Elevated levels of thymidylate synthase (TS) are correlated with resistance to 5 FU and a poor clinical outcome [16-19].

Conventional regimens for treating cancer patients with chemotherapy do not account for interpatient variability in the expression of particular target genes. Such variability results in unpredictable tumor responses and host toxicity. This hospital-based study investigates the role of multiple genetic polymorphisms of six metabolizing and DNA-repair genes (*GSTP1*, Glutathione S-transferase P1; *ABCB1*, multidrug resistance 1; *MTHFR*, methylenetetrahydrofolate reductase; *TYMS*, thymidylate synthase; *ERCC2*, excision repair cross-complementing rodent repair deficiency, complementation group 2; *XRCC1*, X-ray cross-complementing 1) in literature, genomic databases, and the Medline database [10-12,20-23]. The correlations between single nucleotide polymorphisms of the six candidate genes and clinicopathological features of 201 Taiwanese CRC patients, in addition to the relationship between genetic variants and post-therapy early relapse, were also analyzed to elucidate the roles of genotypes of these six genes as predictors of response of CRC patients following 5 FU/LV chemotherapy. Furthermore, this study reviewed current literature regarding the distribution of these six candidate genes and their genotypes in CRC patients of different ethnic groups, and compared differences between Taiwanese CRC patients and those of other races.

This is the first investigation of clinical outcome using multiple chemotherapeutic drug-related genetic polymorphisms for Taiwanese patients with advanced CRC. The ability to predict with a high degree of accuracy which patients are likely to respond to treatment and those who are unlikely to respond will significantly influence the design of new treatment regimens.

## Methods

### Patients and specimens

Enrolled in this prospective study were 201 Taiwanese high-risk International Union Against Cancer (UICC) stage II and stage III CRC patients (median age, 62.09 ± 12.67 years) who were admitted to the Department of Surgery at Kaohsiung Medical University Hospital for elective surgery. Patients with other malignant diseases in their medical history were excluded. All 201 patients underwent radical resection for a primary lesion. Radical resection was defined as any gross residual tumor that did not remain in the surgical bed, and the surgical resection margin is pathologically negative for tumor invasion. All patients were diagnosed as either high-risk stage II (T2 and 3 N0 M0) or III (any T N1 and 2 M0). Patients with risk factors for relapse (tumor poorly differentiated, tumor perforation, number of lymph nodes examined <12 or lymphatic/vascular invasion) were considered high-risk stage II cases. Written informed consent was obtained from all subjects and/or guardians for use of their blood samples. The follow-up endpoint was June 2007. Sample acquisition and subsequent use were approved by the institutional review board at the Kaohsiung Medical University Hospital. Patients were administered six 8-week cycles of adjuvant chemotherapy. Each cycle consisted of leucovorin 500 mg/m^2 ^administered as a 2-h infusion and given weekly for six doses, and 5 FU 500 mg/m^2 ^administered as an intravenous bolus 1 h after the start of leucovorin infusion and repeated weekly for 6 doses. This cycle was then repeated after a 2-week rest period. Postoperative surveillance consisted of comprised medical history, physical examination, and laboratory studies, including assessing serum carcinoembryonic antigen (CEA) levels at 3-month intervals. Abdominal ultrasonography or computed tomography was performed at 6-month intervals, and chest radiography, bone scans, and total colonoscopy were performed annually. Patients were followed up at 3-month intervals for 2 years and at 6-month intervals thereafter. Median follow-up time was 34.2 months (range, 24–40 months). Clinical stage and pathological features of primary tumors were defined according to criteria of the American Joint Commission on Cancer/International Union Against Cancer (AJCC/UICC) [24].

Development of new post-operative recurrent or metastatic lesions was defined as postoperative relapse. Early relapse was defined as local recurrence (tumor growth restricted to the anastomosis or the region of the primary operation) or distant metastasis (distant metastasis or diffuse peritoneal seeding) within 1 year following radical resection.

### DNA extraction

Constitutional gene polymorphisms were analyzed via DNA extraction from 4 ml peripheral blood using a PUREGENE^® ^DNA Isolation Kit (Gentra Systems, Inc., Minneapolis, MN, USA).

### PCR-RFLP

**Figure 1:**
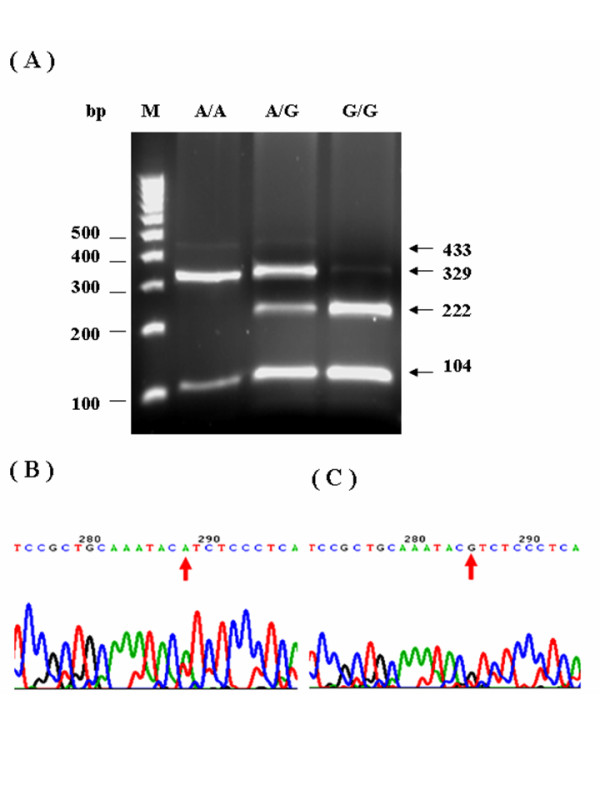
**PCR-RFLP analysis and automated sequencing of *GSTP1 *A313G**. Figures 1A-C present PCR-RFLP analysis results for *GSTP1 *single nucleotide polymorphisms. The *GSTP1 exon5 A313G *was restricted by BsmAI. Digestion resulted in 329 and 104 bp fragments for the A allele, and 222, 107, and 104 bp fragments for the G allele (Fig. 1A: The RFLP of *GSTP1 *results. Fig. 1B: Picture of *GSTP1 *313AA sequences. Fig. 1C: Picture of *GSTP1 *313GG sequences)

**Figure 2:**
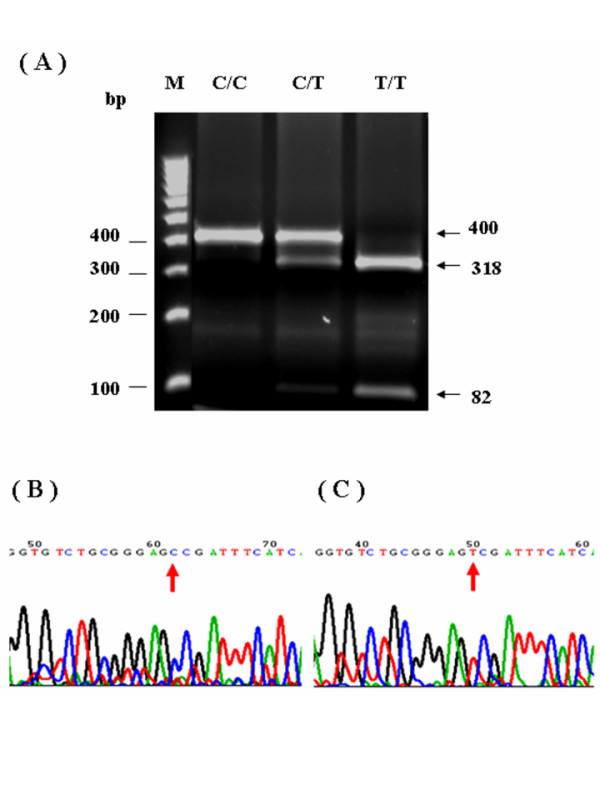
**PCR-RFLP analysis and automated sequencing of *MTHFR *C667T**. Figures 2A-C present PCR-RFLP analysis results for *MTHFR *single nucleotide polymorphisms. Notably, *MTHFR *was restricted by *Hinf*I. Digestion resulted in a 400-bp fragment for the C allele, and 318 and 82 bp fragments for the T allele. (Fig. 2A: Results for RFLP of *MTHFR*. Fig. 2B: Picture of *MTHFR *677CC sequences. Fig. 2C: Picture of *MTHFR *677TT sequences)

**Figure 3:**
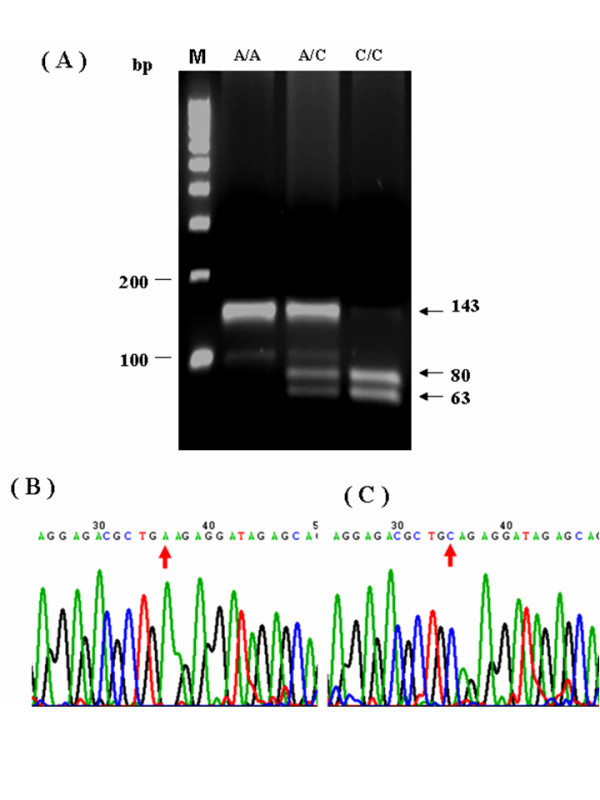
**PCR-RFLP analysis and automated sequencing of *ERCC2 *A2251C**. Figures 3A-C present PCR-RFLP analysis results for *ERCC2 *single nucleotide polymorphisms. Notably, *ERCC2 *was restricted by *Msp*I. Digestion resulted in 143 and 6 bp fragments for the A allele, and 80, 63, and 6 bp fragments for the C allele. (Fig. 3A: Result for RFLP of *ERCC2*. Fig. 3B: Picture of *ERCC2 *2251AA sequences. Fig. 3C: Picture of ERCC2 2251CC sequences)

**Figure 4:**
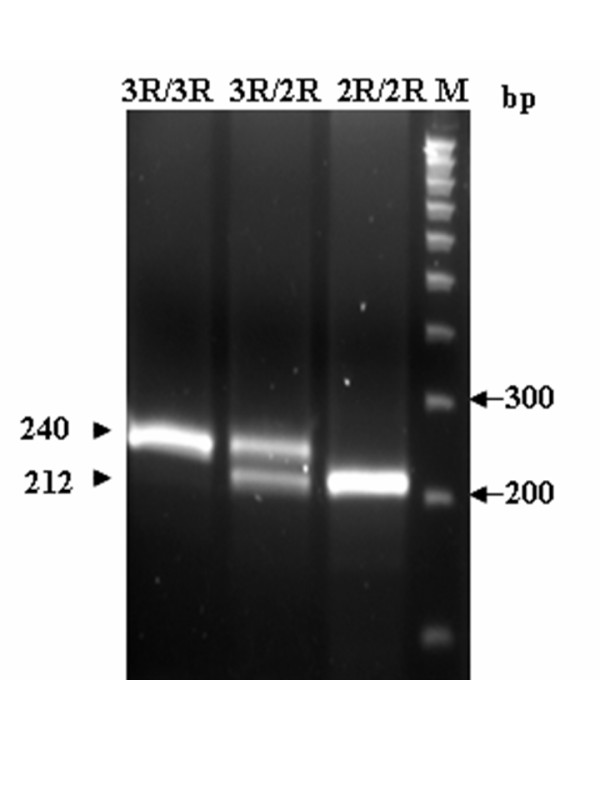
**PCR-RFLP analysis of *TYMS *repeat polymorphisms**. Figure 4 presents PCR-RFLP analysis results for *TYMS *repeat polymorphisms. A 240-bp product was amplified for the 3R allele and a 212-bp product was amplified for the 2R allele.

All genomic DNA from patients were examined using the polymerase chain reaction-restriction fragment length polymorphism (PCR-RFLP) approach to determine the genotypes of *GSTP1*, *ABCB1*, *MTHFR*, *TYMS*, *ERCC2*, and *XRCC1*. Following digestion with suitable restriction enzymes, PCR fragments were separated on a 2.5–3.0% agarose gel and visualized after ethidium bromide staining. This study also utilized the automated sequencing approach to confirm PCR-RFLP results (Figs. 1, 2, 3, 4). All primers utilized in this study were designed by using Primer3 freeware [25]. Table 1 presents the primer sequences and restriction enzymes. The PCR reaction volume was 40 μL. The PCR conditions, for the *GSTP1 *polymorphism were as follows: 95°C for 5 min, 35 cycles at 95°C for 30 s, annealing at 65°C for 10 s, and 72°C for 25 s. A 433-bp product was amplified following digestion with *BsmAI*. Digestion generated fragments of 329 and 104 bp for the A allele, and fragments of 222, 107, and 104 bp for the Gallele. The PCR conditions for the *ABCB1 *polymorphism were as follows: 95°C for 5 min, 35 cycles at 95°C for 30 s, annealing at 58°C for 15 s, and 72°C for 20 s. A 244-bp product was amplified following digestion with *Dpn*II. Digestion generated fragments of 172 and 72 bp for the C allele, and a 244-bp fragment for the T allele. The PCR conditions for the *MTHFR *polymorphism were as follows: 95°C for 5 min, 35 cycles at 95°C for 30 s, annealing at 70°C for 20 s, and 72°C for 25 s. A 400-bp product was amplified following digestion with *Hinf*I. Digestion produced a 400-bp fragment for the C allele, and 318 and 82 bp fragments for the T allele. The PCR conditions for the *TYMS *polymorphism were as follows: 95°C for 5 min, 35 cycles at 95°C for 30 s, and 72°C for 35 s. A 240- and 212-bp product was amplified for the 3R allele and 2R allele, respectively. The PCR conditions for the *ERCC2 *polymorphism were as follows: 95°C for 5 min, 35 cycles at 95°C for 30 s, annealing at 64°C for 10 s, and 72°C for 20 s. A 149-bp product was amplified following digestion with *Pst*I. Digestion generated 143- and 6-bp fragments for the A allele, and fragments of 80, 63, and 6 bp for the C allele. The PCR conditions for the *XRCC1 *polymorphism were as follows: 95°C for 5 min, 35 cycles at 95°C for 30 s, annealing at 62°C for 20 s, and 72°C for 20 s. A 208-bp product was amplified following digestion with *Msp*I. Digestion produced 156- and 52-bp fragments for the G allele, and a 208-bp fragment for the A allele.

**Table 1:** Characteristics of the studied polymorphisms with primer sequences and restriction enzymes

Gene	Primer sequences	Restriction enzyme	Polymorphism
*GSTP1*	[F]:G5'-GTAGTTTGCCCAAGGTCAAG-3' [R]:G5'-AGCCACCTGAGGGGTAAG-3'	*BsmAI*	Ile105Val (313A>G)
*ABCB1*	[F]:G5'-GATCTGTGAACTCTTGTTTTC-3' [R]:G5'-GAAGAGAGACTTACATTAGGC-3'	*DpnII*	Ile1145Ile (3435C>T)
*MTHFR*	[F]:G5'-CTTGAACAGGTGGAGGCCAGC-3' [R]:G5'-AGGACGGTGCGGTGAGAGTG-3'	*HinfI*	Ala222Val (677C>T)
*TYMS*	[F]:G5'-GACCCCGCCGAGCAGGAAGA-3' [R]:G5'-GTGCCCGTGCGGTCGTCCTT-3'	-	28-bp repeat (5'-UTR)
*ERCC2*	[F]:G5'-TCTGCAGGAGGATCAGCTG-3' [R]:G5'-GCAAGACTCAGGAGTCAC-3'	*PstI*	Lys751Gln (2251A>C)
*XRCC1*	[F]:G5'-TTGTGCTTTCTCTGTGTCCA-3' [R]:G5'-TCCTCCAGCCTTTTCTGATA-3'	*MspI*	Arg399Gln (1196G>A)

### Statistical analysis

All data were analyzed using the Statistical Package for the Social Sciences Version 12.0 (SPSS, Inc., Chicago, IL, USA). The correlation between a polymorphism and relapse status was assessed using the relative risk ratio and a 95% confidence interval (CI). This is an exploratory and hypothesis-generating study. To clarify the clinical significance of these combined genotypes as predictors of postoperative relapse, a multivariate adjustment was performed by logistic regression analysis. The two-sided Pearson χ^2 ^test was applied to assess the differences in distributions of genotypes between different races. A value of *P *< 0.05 was considered statistically significant.

## Results

### Patient characteristics

In total, 201 patients were enrolled. Of these, 118 were male (58.7%) and 83 were female (41.3%). Thirty-three patients (16.4%) were aged : ≦50 years and 120 (59.7%) were >60 years (range, 33–75 years). Thirty cases (14.9%) developed early relapse, either local recurrence or distant metastasis, during follow-up. Primary tumor location for 146 cases (72.6%) was the colon and 55 (27.4%) was the rectum. In total, 105 cases (52.2%) were UICC stage II, and 96 cases (47.8%) were UICC stage III. Table 2 lists the other clinicopathological characteristics of patients and tumors.

**Table 2:** Correlations between clinicopathological features and recurrence status for 201 postoperative colorectal cancer patients

Characteristics	Total cases N (%)	Early recurrence N (%)	Non-early recurrence N (%)	*P*
Gender				
Male	118 (58.7)	17 (56.7)	101 (59.1)	0.806
Female	83 (41.3)	13 (43.3)	70 (40.9)	
Age (years)				
>60	120 (59.7)	23 (76.7)	97 (56.7)	0.105
51~60	48 (23.9)	5 (16.7)	43 (25.1)	
≦ 50	33 (16.4)	2 (6.6)	31 (18.2)	
Tumor Size				
<5 cm	107 (53.2)	16 (53.3)	91 (53.2)	0.991
≧ 5 cm	94 (46.8)	14 (46.7)	80 (46.8)	
Location				
Colon	146 (72.6)	22 (73.3)	124 (72.5)	0.926
Rectum	55 (27.4)	8 (27.7)	47 (27.5)	
Depth of tumor invasion				
T2	26 (12.9)	0 (0)	25 (14.6)	0.025
T3 + T4	175 (87.1)	30 (100)	146 (85.4)	
Lymph node metastasis				
Negative	110 (54.7)	10 (33.3)	100 (58.5)	0.011
Positive	91 (45.3)	20 (66.7)	71 (41.5)	
Stage (UICC)^a^				
II	105 (52.2)	9 (30)	96 (56.1)	0.008
III	96 (47.8)	21 (70)	75 (43.9)	
Histology				
Well + moderately differentiated	170 (84.6)	23 (76.7)	147 (86)	0.193
Poorly differentiated	31 (15.4)	7 (23.3)	24 (14)	

^a^International Union Against Cancer

### Correlation between early relapse and clinicopathological data

No statistical correlations existed between early relapse status and sex, age, tumor size, tumor location and histology (*P *> 0.05; Table 2). However, depth of tumor invasion, lymph node metastasis and cancer stage were significantly related to early relapse (*P *< 0.05). Risk of early relapse was higher in T3+T4 that had a deeper invasion than did T2 (OR, 1.205; 95% CI, 1.127–1.289; *P *= 0.025). Stage III patients had a higher early relapse rate than stage II patients (OR, 2.897; 95% CI, 1.243–6.381; *P *= 0.011), and early relapse was higher in cases with lymph node metastasis than those with no lymph node metastasis (OR, 2.987; 95% CI, 1.293–6.899; *P *= 0.008).

### Comparison of genotype distributions between Taiwanese CRC patients and those of other races

To compare the genotypes of Taiwanese CRC patients with those of other races (Table 3), this study compared the distribution of gene polymorphisms, such as *GSTP1 *313A>G (AA, AG, GG), *ABCB1 *3435C>T (CC, CT, TT), *MTHFR *677C>T (CC, CT, TT), *TYMS *tandem repeat (3R3R, 2R3R, 2R2R), *ERCC2 *2251A>C (AA, AC, CC) and *XRCC1 *1196G>A (GG, GA, AA), in various races, races – Caucasian, Japanese, Korean, Chinese, and Taiwanese. This study also included our findings from a literature review.

**Table 3:** Comparison of genotype and allele frequencies (%) between Taiwanese colorectal cancer patients and those of other ethnic groups

Genotype	Caucasian	Japanese	Korean	Chinese	Taiwanese	Study data
*GSTP1*	*Van der Logt*^26^				*Yeh*^27^	
AA	42.0	-	-	-	67.9	64.2
AG	47.4	-	-	-	28.7	32.8
GG	10.6	-	-	-	3.4	3.0
A allele	0.657	-	-	-	0.823	0.807
G allele	0.343	-	-	-	0.177	0.193
*ABCB1*	*Kurzawski*^28^	*Komoto*^29^	*Bae*^30^			
CC	22.3	29.2	28.8	-	-	34.8
CT	44	58.3	56.8	-	-	51.8
TT	33.7	12.5	14.4	-	-	13.4
C allele	0.443	0.584	0.572	-	-	0.607
T allele	0.557	0.416	0.428	-	-	0.393
*MTHFR*	*Curtin*^31^	*Yin*^32^	*Kim*^33^			
CC	46	39.4	35.4	-	-	60.7
CT	45	48.2	50.2	-	-	35.8
TT	9	12.4	14.4	-	-	3.5
C allele	0.685	0.635	0.605	-	-	0.786
T allele	0.315	0.365	0.395	-	-	0.214
*TYMS*	*Chen*^36^	*Kawakami*^34^		*Marsh*^35^		
3R/3R	32	69	-	67	-	67.2
2R/3R	50	27	-	31	-	29.3
2R/2R	18	4	-	2	-	3.5
3R allele	0.570	0.825	-	0.825	-	0.818
2R allele	0.430	0.175	-	0.175	-	0.182
*ERCC2*	*Stoehlmacher*^37^				*Yeh*^38^	
AA	25	-	-	-	84	87.6
AC	61	-	-	-	15.6	11.9
CC	14	-	-	-	0.4	0.5
A allele	0.555	-	-	-	0.918	0.935
C allele	0.445	-	-	-	0.082	0.065
*XRCC1*	*Stoehlmacher*^37^		*Hong*^39^		*Yeh*^38^	
GG	39	-	53.6	-	56.7	61.7
GA	52	-	42.1	-	36.2	31.8
AA	9	-	4.3	-	7.1	6.5
G allele	0.650	-	0.747	-	0.748	0.776
A allele	0.350	-	0.253	-	0.252	0.224

NOTE: *author*^*n *^in this table is designated by a reference number (^*n*^) and the abbreviated name of the first author in the previously paper.

For *GSTP1*, 129 (64.2%) were AA, 66 (32.8%) were AG and 6 (3.0%) were GG genotype carriers, as compared with Caucasians [26] (AA, 42%; AG, 47.4%; GG, 10.6%) and Taiwanese [27] (AA, 67.9%; AG, 28.7%; GG, 3.4%). The distribution of *GSTP1 *313A>G genotypes in our data differed significantly from that of Caucasian patients (*P *= 0.003); however, no significant differences existed when compared with previous Taiwanese studies (*P *= 0.827).

For *ABCB1*, 70 (34.8%) were CC, 104 (51.8%) were CT and 27 (13.4%) were TT genotype carriers, as compared with Caucasians [28] (CC, 22.3%; CT, 44%; TT, 33.7%), Japanese [29] (CC, 29.2%; CT, 58.3%; TT, 12.5%) and Korean [30] (CC, 28.8%; CT, 56.8%; TT, 14.4%). The distribution of *ABCB1 *3435C>T genotypes was significantly different from that in Caucasians (*P *= 0.001) and not significantly different from that in Japanese (*P *= 0.641), and Korean populations (*P *= 0.661).

For *MTHFR*, 122 (60.7%) were CC, 72 (35.8%) were CT and 7 (3.5%) were TT genotype carriers, as compared with Caucasians [31] (CC, 46%; CT, 45%; TT, 9%), Japanese [32] (CC, 39.4%; CT, 48.2%; TT, 12.4%), and Korean [33] (CC, 35.4%; CT, 50.2%; TT, 14.4%) populations. Significantly different distributions of *MTHFR 677C>T *(*CC, CT, TT*) genotypes existed when our data was compared with Japanese (*P *= 0.005) or Korean data (*P *= 0.001), and it does not significantly different from Caucasian data (*P *= 0.081).

For *TYMS*, 135 (67.2%) were 3R/3R, 59 (29.3%) were 2R/3R, and 7 (3.5%) were 2R/2R genotype carriers, as compared with Caucasians (3R/3R, 32%; 3R/2R, 50%; 2R/2R, 18%), Japanese [34] (3R/3R, 69%; 3R/2R, 27%; 2R/2R, 4%), and Chinese [35] (3R/3R, 67%; 3R/2R, 31%; 2R/2R, 2%) populations. The highest expression of *TYMS *3R/3R in this study of Taiwanese patients was similar to that of Japanese (*P *= 0.951) and Chinese populations (*P *= 0.693), but different from that of the Caucasian population [36] (*P *< 0.0001).

For *ERCC2*, 176 (87.6%) were AA, 24 (11.9%) were AC, and 1(0.5%) were CC genotype carriers, as compared with Caucasians [37] (AA, 25%; AC, 61%; CC, 14%) and Taiwanese [38] (AA, 84%; AC, 15.6%; CC, 0.4%) populations. The highest expression of *ERCC2 *AA in this study was similar to that in a previous study [38] (*P *= 0.436) and different from that of a Caucasian population (*P *< 0.0001). Caucasian CRC cancer patients presented with *ERCC2 *AC as the most common polymorphism genotype. In this study and another previous Taiwanese studies, *ERCC2 *2251A>C AA was more common than AC and CC, while another study indicated that *ERCC2 *2251A>C AC was the most frequent genotype.

For *XRCC1*, 124 (61.7%) were GG, 64 (31.8%) were GA and 13 (6.5%) were AA genotype carriers, as compared with Caucasian [37] (GG, 39%; GA, 52%; AA, 9%), Korean [39] (GG, 53.6%; GA, 42.1%; AA, 4.3%), and Taiwanese [38] (GG, 56.7%; GA, 36.2%; AA, 7.1%) populations. The highest expression of *XRCC1 *GG in this study was similar to that for Koreans (*P *= 0.257) and other Taiwanese studies (*P *= 0.802), but different from that of the Caucasian population (*P *= 0.006).

### Correlation between genetic polymorphisms with clinicopathological data

This study assessed correlations between genetic polymorphisms and clinicopathological features of 201 Taiwanese CRC patients (Table 4). No statistically significant correlations existed between genotype distributions and sex, age, tumor site or location, depth of tumor invasion, lymph node metastasis, cancer stage, or histology (all *P *> 0.05).

**Table 4:** Correlation between gene polymorphism and clinicopathological properties of 201 postoperative colorectal cancer patients

		*GSTP1*		*ABCB1*		*MTHFR*		*TYMS*		*ERCC2*		*XRCC1*	
Characteristics	Total cases (n)	AA	AG or GG	CC	CT or TT	CC	CT or TT	3R3R	2R3R or 2R2R	AA	AC or CC	GG	GA or AA
Gender													
Male	118	73	45	46	72	68	50	79	39	102	16	73	45
Female	83	56	27	24	59	54	29	56	27	74	9	51	32
		*P *= 0.414		*P *= 0.140		*P *= 0.228		*P *= 0.938		*P *= 0.566		*P *= 0.952	
Age (yr)													
>60	120	76	44	39	81	76	44	80	40	102	18	74	46
51~60	48	28	20	20	28	27	21	32	16	42	6	33	15
≤50	33	25	8	11	22	19	14	23	10	32	1	17	16
		*P *= 0.293		*P *= 0.520		*P *= 0.643		*P *= 0.944		*P *= 0.182		*P *= 0.293	
Tumor Size													
<5 cm	107	67	40	37	70	67	40	72	35	94	13	71	36
≥5 cm	94	62	32	33	61	55	39	63	31	82	12	53	41
		*P *= 0.622		*P *= 0.938		*P *= 0.552		*P *= 0.968		*P *= 0.895		*P *= 0.147	
Location													
Colon	146	96	50	52	94	86	60	96	50	127	19	89	57
Rectum	55	33	22	18	37	36	19	39	16	49	6	35	20
		*P *= 0.448		*P *= 0.701		*P *= 0.397		*P *= 0.488		*P *= 0.687		*P *= 0.728	
Depth of tumor invasion													
T2	26	19	7	9	17	15	11	17	9	22	4	18	8
T3 + T4	175	110	65	61	114	107	68	118	57	154	21	106	69
		*P *= 0.311		*P *= 0.981		*P *= 0.737		*P *= 0.836		*P *= 0.626		*P *= 0.397	
Lymph node metastasis													
Negative	110	68	42	41	69	65	45	75	35	95	15	66	44
Positive	91	61	30	29	62	57	34	60	31	81	10	58	33
		*P *= 0.443		*P *= 0.423		*P *= 0.608		*P *= 0.736		*P *= 0.571		*P *= 0.588	
Stage^a^													
II	105	64	41	41	64	62	43	71	34	91	14	63	42
III	96	65	31	29	67	60	36	64	32	85	11	61	35
		*P *= 0.318		*P *= 0.189		*P *= 0.617		*P *= 0.886		*P *= 0.687		*P *= 0.606	

^a^International Union Against Cancer

### Correlation between early relapse and genetic polymorphisms

The correlations between gene polymorphisms (*GSTP1 *313A>G, *ABCB1 *3435C>T, *MTHFR *667C>T, *TYMS *double or triple tandem repeats, *ERCC2 *2251A>C and *XRCC1 *1196G>A) and patients with or without early recurrence were examined. Statistical analyses indicate that genotype polymorphisms of *ERCC2 *were strongly correlated between patients with recurrent and non-recurrent tumors (*P *= 0.006; Table 5); however, *GSTP1*, *ABCB1*, *MTHFR*, *TYMS*, and *XRCC1 *genotypes polymorphisms were not correlated (*P *> 0.05; Table 5). The Taiwanese CRC patients with *ERCC2 *2251AC and *ERCC2 *2251CC genotypes have a risk of recurrence 3.294 times greater than that of those with other genotypes (*P *= 0.01; 95% CI, 1.272–8.532).

**Table 5:** Distribution of gene polymorphisms in 201 colorectal cancer patients and regarding the status of recurrence after surgery

Genotype	Total cases N (%)	Early Recurrence N (%)	Non-early recurrence N (%)	*P*
*GSTP1 *Ile105Val (313A>G)				
AA	129 (64.2)	19 (63.4)	110 (64.3)	0.990
AG	66 (32.8)	10 (33.3)	56 (32.7)	
GG	6 (3.0)	1 (3.3)	5 (3)	
*ABCB1 *Ile1145Ile (3435C>T)				
CC	70 (34.8)	10 (33.3)	60 (35.1)	0.853
CT	104 (51.8)	15 (50)	89 (52)	
TT	27 (13.4)	5 (16.7)	22 (12.9)	
*MTHFR *Ala222Val (677C>T)				
CC	122 (60.7)	18 (60)	104 (60.8)	0.994
CT	72 (35.8)	11 (36.7)	61 (35.7)	
TT	7 (3.5)	1 (3.3)	6 (3.5)	
*TYMS *double (2R) or triple (3R) tandem repeat				
3R/3R	135 (67.2)	20 (66.7)	115 (67.2)	0.492
2R/3R	59 (29.3)	10 (33.3)	49 (28.7)	
2R/2R	7 (3.5)	0 (0)	7 (4.1)	
*ERCC2 *Lys751Gln(2251A>C)				
AA	176 (87.6)	22 (73.4)	154 (90.1)	0.006
AC	24 (11.9)	7 (23.3)	17 (9.9)	
CC	1 (0.5)	1 (3.3)	0 (0)	
*XRCC1 A*rg399Gln(1196G>A)				
GG	124 (61.7)	17 (56.7)	107 (62.6)	0.654
GA	64 (31.8)	10 (33.3)	54 (31.6)	
AA	13 (6.5)	3 (10)	10 (5.8)	

## Discussion

This is an exploratory and hypothesis-generating study. This study attempted to move beyond single genetic polymorphisms to a more comprehensive investigation that identifies genomic variants and patterns and performed early relapse analysis. This study investigated six functional genomic polymorphisms in genes, which have different enzyme functions, or expressions, and DNA repair by PCR-RFLP assay and cycling sequencing. This is the first comprehensive study to investigate genotype frequencies of six gene polymorphisms in Taiwanese CRC patients. Additionally, this study analyzed the genotypes of 201 Taiwanese CRC patients and demonstrated that genotype distributions have ethnic variations.

In 5FU-related genes, the incidence of the *TYMS *gene promoter 3R/3R genotype in the 201 cases in this study is similar to that in Japanese [34] and Chinese [35] populations, and higher than that in Caucasian populations [36]. Neither the *TYMS *2R allele nor the *TYMS *3R allele is predominant in Caucasians; however, the *TYMS *3R allele is predominant in Asian populations. For the *MTHFR *C677T polymorphism, the incidence of the 677CC genotype in this Taiwanese CRC population was higher than that for Caucasians [31], and Japanese [32], and Korean [33] populations (Table 3). Notably, the *C *allele and *T *allele frequencies in Caucasian and Asian populations are similar.

This study assessed the *ABCB1 *3435C>T of 201 Taiwanese CRC patients. The frequency of the TT genotype in the 201 Taiwanese patients is similar to that in Japanese [29] and Korean [30] patients, but lower than that in Caucasian populations [28]. The allele frequency of C and T did not differ between Caucasians and Asians.

The genotype data for cell detoxification-related gene polymorphism, *GSTP1 *313A>G, are similar to that for 408 Taiwanese CRC patients in a study by Yen *et al*. [27]. Caucasian populations have an equal incidence of AA homozygotes and AG heterozygotes [26]; however, the Taiwanese population has more AA homozygotes than AG heterozygotes. The allele frequency of Ile is also more predominant in Taiwanese patients than in Caucasian patients.

Since the genetic polymorphisms of DNA-repair enzymes may influence DNA adduct levels [40-42], the particular degree of DNA repair capacity can be utilized to identify genetically high-risk individuals for human cancers [43]. Resistance to platinum agents has been attributed to increased tolerance to platinum DNA adducts, enhanced DNA repair, or decreased drug accumulation [44]. Proteins of the nucleotide excision repair (NER) pathway, in particular, are believed play a central role in repair of DNA damage caused by platinum compounds. The enzymes related to the DNA repair system, *XRCC1 *and *ERCC2*, were similar to those identified in other studies of Taiwanese CRC patients [38]. Although very few studies have investigated these two polymorphisms in Asian CRC populations, the incidence of *XRCC1 *G1196A in a Korean population [39] is similar to that in the 201 Taiwanese patients in this study. However, the frequency of the *XRCC1 *GG homozygous and GA heterozygous patients in Caucasian [37] and Asian populations differ significantly. Moreover, the frequency of the G allele and A alleles do not differ between Caucasians and Asians. For *ERCC2 *2251A>C, the genotype and allele frequencies are dissimilar between Taiwanese and Caucasians [37]. Neither the A allele nor C allele has been identified as predominant in Caucasians, whereas the A allele is predominant in the Taiwanese (Table 3).

In 2004, Shirao *et al. *compared the efficacy, toxicities, and pharmacokinetics of an oral regimen consisting of uracil/tegafur (UFT) and LV between Japanese patients and Caucasian patients in the United States [45]. Although the response rate did not differ (36.4% for Japanese patients and 34.1% for patients in the United States), a difference existed in toxicity profile, specifically the incidence of diarrhea – 9% in the Japanese population and 22% in the Caucasian population in the United States. Since genotype frequencies were similar between Japanese and Taiwanese populations, we suggest that cancer chemotherapy efficacy, toxicities, and pharmacokinetics would be similar to those for Japanese patients. Consequently, we hypothesize that these polymorphisms screened have the ability to predict toxicity, clinical outcome and survival in Taiwanese CRC patients. This hypothesis and suggestion warrant further investigation.

Studies focusing on acute relapse risk of CRC and these metabolizing and DNA-repair genes are limited and controversial. In addition to the statistically significant role of early relapse, including depth of tumor invasion (*P *= 0.025), lymph node metastasis (*P *= 0.011), and cancer stage (*P *= 0.008), analytical results in this study indicate *ERCC2 *2251A>C AC and CC gene polymorphismswere statistically significant in predicting early relapse.

The DNA repair systems play an important role in maintaining genomic integrity and preventing carcinogenesis [46]. At least four pathways for DNA repair operate on specific DNA damage types [47]. Nucleotide excision repair is the primary pathway in humans. The *ERCC2 *gene plays a dominant role in nucleotide excision repair and basal transcription, both of which are crucial to the elimination of bulky DNA adducts. Furthermore, the *ERCC2 *protein is essential for nucleotide excision repair activity [48,49]. The *ERCC2 *gene consists of 23 exons at 19q13.3. Several single nucleotide polymorphisms have been identified in the coding part of *ERCC2*, of which Ile199Met (C/G), His201Tyr (C/T), Asp312Asn (G/A), and Lys751Gln (A/C) result in amino acid changes; however, codon Arg156Arg (C/A) and Asp711Asp (C/T) are silent polymorphisms. The functional effect of *ERCC2 *polymorphisms remains unclear. The polymorphisms at codon 312 and 751 have been analyzed extensively for their potential ability to increase lung cancer risk [49,50]. Although an earlier study showed that *ERCC2 *homozygous variant individuals were at increased risk of adenomatous polyps [51], associations between DNA repair gene polymorphisms and CRC have not been explored extensively. This study suggests that CRC patients who have the *ERCC2 *2251AC (751Lys/Gln) or 2251CC (751Glu/Gln) genotypes have a significantly increased early relapse risk (OR = 3.294; 95% CI, 1.272–8.532), and no statistically significant correlation exists between genotype distributions and clinicopathological features (all *P *> 0.05).

This study examined potential genetic predictors of CRC relapse. A recent study [52] determined that the C(Glu) allele of the ERCC2 Lys751Gln variant allele marginally increased lung cancer risk (OR = 3.61, P = 0.04) in a Chinese population, and another identified [53] increased risk for squamous cell carcinoma of the head and neck in patients with the Gln/Gln genotype when compared with the Lys/Lys group. Experimental results in this study indicate that the *ERCC2 *2251A>C AC and CC gene polymorphisms can predict early relapse; these experimental results are in agreement with the risk role of the C (Glu) allele of ERCC2 Lys751Gln in early relapse. Inherited single nucleotide polymorphisms of DNA repair genes may contribute to variations in DNA repair capacity and susceptibility to cancer. The molecular functional effect of ERCC2 polymorphisms remains unclear. Although some researchers have identified incomplete repair of DNA damage or of aromatic DNA adducts in the presence of ERCC2 variant alleles [42,54-58], we hypothesize that the malignant entity of the C (Glu) allele of ERCC2 Lys751Gln is responsible for poor prognosis of early relapse among CRC patients receiving similar chemotherapeutic regimens in this study. In 2001, Park reported the finding of a significant relationship between clinical response to chemotherapy (combined oxaliplatin and 5FU) and ERCC2 Lys751Gln polymorphisms. Patients with the Gln/Gln genotype had significantly shorter survival or increased relative risk of dying when compared with the Lys/Lys group [10]. These experimental results suggest that *ERCC2 *2251A>C may indeed be of functional importance in Taiwanese CRC. Aggressive chemotherapy or combined radiotherapy will be considered for patients with this poor prognostic genotype.

## Conclusion

In conclusion, analytical data in this study suggest that the polymorphism *ERCC2 *A2251C is associated with risk of CRC early relapse in a Taiwanese population. Additionally, this study analyzed six gene polymorphisms associated with the clinical outcome of Taiwanese CRC patients. These genotypic frequencies vary among ethnic groups. Studies with relatively larger sample sizes are needed to elucidate the effects of these polymorphisms on early relapse risk in this population. However, experimental results in this study serve as a basis for large-scale correlational studies on the relevance of these variants in predicting early relapse of CRC in a Taiwanese population.

## Abbreviations

CRC, colorectal cancer; *GSTP1*, Glutathione S-transferase P1; *ABCB1*, multidrug resistance 1; *MTHFR*, methylenetetrahydrofolate reductase; *TYMS*, thymidylate synthase; *ERCC2*, excision repair cross-complementing rodent repair deficiency, complementation group 2; *XRCC1*, X-ray cross-complementing1; PCR-RFLP, polymerase chain reaction restriction fragment length polymorphism.

## Competing interests

The author(s) declare that they have no competing interests.

## Authors' contributions

MYH analyzed the data and wrote the manuscript. WYF, SCL and TLC made substantial contributions in data acquisition, molecular genetic analyses, statistical analyses and data interpretation, and helped in manuscript preparation. JYW and SRL participated in study design and coordination. All authors read and approved the final manuscript.

## Pre-publication history

The pre-publication history for this paper can be accessed here:


